# Occurrence of severe rotavirus gastroenteritis in children younger than three years of age before and after the introduction of rotavirus vaccine: a prospective observational study in four pediatric clinics in Shibata City, Niigata Prefecture, Japan

**DOI:** 10.1080/21645515.2020.1720435

**Published:** 2020-07-01

**Authors:** Tomohiro Oishi, Masamichi Matsunaga, Tokushi Nakano, Shoji Sudo, Hiroaki Kuwajima, Shuko Tokuriki, Shibata RVGE Study

**Affiliations:** a Department of Pediatrics, Kawasaki Medical School, Kurashiki, Japan; b Pediatric Department, Niigata Prefectural Shibata Hospital, Shibata City, Japan; c Pediatric Department, Nakano Children’s Clinic, Shibata City, Japan; d Pediatric Department, Sudo Pediatric Clinic, Shibata City, Japan; e Pediatric Department, Kuwajima Clinic, Shibata City, Japan; f Pediatric Department, Twin Smile Clinic, Shibata City, Japan

**Keywords:** Rotavirus, vaccine, gastroenteritis, Japan, clinic, coverage rate, children

## Abstract

In Japan, rotavirus (RV) vaccines have already been introduced but not used for universal vaccination as of 2018. Therefore, we identified cases of severe rotavirus gastroenteritis (RVGE) in children younger than three years of age and investigated the occurrence of infection before and after the introduction of RV vaccines. An ecological study through prospective surveillance was conducted in four pediatric clinics in Shibata City, Niigata Prefecture, Japan, during the 2011 to 2018 RVGE epidemic seasons. We divided the study period into three eras: pre-vaccine introduction era (2011), low-mid coverage transitional era (2012 to 2014, RV vaccine coverage rate: 32.9–56.5%), and high coverage plateau era (2015 to 2018, 67.7–81.7%). In this study, the incidence rate of severe RVGE was significantly lower in the plateau era than in the pre-vaccine introduction and transitional eras. Furthermore, the hospitalization rate due to RVGE in Shibata City was lower in the plateau era than in the pre-vaccination introduction and transitional eras. The number of hospitalizations due to RVGE in subjects who required or did not require intravenous rehydration at the pediatric clinics significantly decreased with the increase in vaccine coverage rates by more than 70% in the plateau era.

## Introduction

Rotavirus (RV) infection is the most common cause of severe diarrhea in children worldwide.^1^ Although fatal cases are rare in Japan, some patients with rotavirus gastroenteritis (RVGE) experience severe dehydration, leading to life-threatening conditions. Currently, two oral RV vaccines, Rotarix® (GlaxoSmithKline Vaccines GSK, Rixensart, Belgium) and RotaTeq® (Merck & Co., Inc., Whitehouse Station, NJ, USA), which prevent RV disease, are approved in more than 100 countries^2^ and are incorporated into the National Immunization Programs (NIP) in 98 countries as of end of 2018.^3^ With the introduction of the RV vaccine, the number of deaths worldwide caused by the virus in children less than five years of age declined from 528,000 in 2000^4^ to 128,500 in 2016.^5^ In Japan, Rotarix and RotaTeq have been in the market since November 2011 and July 2012, respectively.

Previously, a prospective observational study that assessed the impact of the RV vaccination was conducted.^6^ The changes in disease burden overtime, i.e., before and after the introduction of RV vaccines were investigated. Since it is easier to visit a clinic anytime, some patients receive intravenous rehydration to avoid admission. Therefore, the aim of the present study was to evaluate the impact of the RV vaccination on severe RVGE among outpatient settings. The methods we used in the present study were almost the same as those of our previous study during the 2011 and 2014 RV epidemic seasons.^7,8^ However, we included all the results obtained between 2011 and 2018 because RV vaccination was introduced in Japan during this time period.

## Materials and methods

### Study design

We conducted the present ecological study in Shibata City, having a population of about 100,000, and the number of births is approximately 770 babies per year. The collaborators were four pediatricians at primary-care pediatric clinics in Shibata City.

Shibata City has only one hospital with a pediatric ward, i.e., the Niigata Prefectural Shibata Hospital. Therefore, all children living in Shibata City are admitted to this hospital. Additionally, children with acute gastroenteritis (AGE) often visit one of the four well-known pediatric clinics in Shibata City. Thus, almost all pediatric patients in Shibata City were covered by assessing the patients who visited these clinics. A previous review described that severe illness is most common between the age of 6 months and 2 years.^9^ Therefore, the participants of this study were children younger than three years of age who had AGE and visited one of the recruiting clinics in Shibata City from February 2011 to May 2018. Information on date of visit, date of birth, sex, living area, symptoms such as diarrhea and vomiting, and intravenous administration of rehydration fluids were collected for all the eligible children. We defined AGE based on the occurrence of symptoms such as diarrhea and vomiting, lasting not for more than 14 days.^10^ Severe AGE was defined when the patient required intravenous rehydration.

Furthermore, we confirmed the presence of RV antigen in the fecal samples, using ImmunoCard^TM^ ST Rotavirus (Fujirebio Inc, catalog number: 750030) as a rapid fecal rotavirus test. Additionally, patients with severe RVGE were defined as those with severe AGE and with positive RV antigen test results. We collected all the information from the patients in order to evaluate their conditions using the modified Vesikari scale.^11^ Because most patients included were those with AGE, both clinicians and patients did not have enough time to obtain or provide informed consent. Therefore, oral informed consents were obtained from the parents/guardians of the pediatric patients before participating in the study. Only information from the children who fulfilled the inclusion criteria was extracted from medical charts and analyzed.

The number of hospitalized patients with AGE and RVGE at the Niigata Prefectural Shibata Hospital from 2008 to 2018 was retrospectively collected for reference. These data included the number of patients who were not only directly admitted to the Hospital, but also via four pediatric clinics.

Vaccine coverage rates were calculated according to RV vaccination records from the four pediatric clinics, Shibata Hospital, and Tomita gynecologic clinic where some children in Shibata City visit not only for the treatment of their diseases but also for their vaccinations.

We divided the observation study period into three eras. The pre-vaccine introduction era (2008 to 2011) represents the era before the RV vaccine was introduced in Japan. The low-mid coverage transitional era (2012 to 2014, RV vaccine coverage rate: 32.9–56.5%) represents the increasing vaccine coverage rates each year. Finally, the high coverage plateau era (2015 to 2018, 67.7–81.7%) represents the era in which there was no significant increase in vaccine coverage rates.

### Statistical analyzes

To calculate the statistical significance of differences between distributions of categorical variables, we used a Chi-square test. We also used Student’s t-test to compare means of numerical variables. We calculated the incidences and 95% confidence intervals of outpatient severe RVGE, severe AGE, and AGE, based on the number of enrolled patients. We compared these incidences between 2011 and the other years using Poisson regression analysis. Microsoft Excel version 14 was used for statistical analyzes.

### Viral antigen analyzes

We performed genotyping of rotavirus using fecal samples obtained from the patients. Analyses were performed using reverse transcription-polymerase chain reaction at the Niigata Prefectural Institute of Public Health and Environmental Sciences, as previously described.^8^

### Ethical adherence

This study was conducted in compliance with the Ethical Guidelines for Epidemiological Research (Partial revision: December 1, 2008, The Ministry of Education, Culture, Sports, Science and Technology; Ministry of Health, Labor and Welfare), which is based on the Declaration of Helsinki–Ethical Principles for Medical Research Involving Human Subjects, and the Ethics Guidelines for Medical Research for Humans (December 22, 2014, The Ministry of Education, Culture, Sports, Science and Technology; Ministry of Health, Labor and Welfare). Prior to conducting the study, the Ethical Review Committee of the Niigata Prefectural Shibata Hospital reviewed the ethical as well as scientific aspects of the study protocol (e.g., study design, population, time period, etc.) and approved the proposed study.

## Results

**Figure 1.**
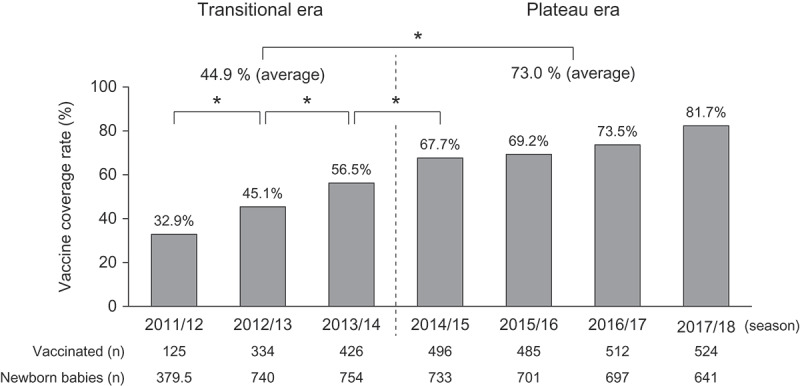
Vaccine coverage rates of RV1 and RV5 in Shibata City by season. Number of infants vaccinated with more than one dose of Rotarix or RotaTeq in the 2011/12 season, between November 25, 2011 (vaccine introduction in Japan) and May 31, 2012. Coverage rate: Proportion of infants vaccinated was calculated based on the annual number of newborn babies in Shibata City. However, the period of research was from November 25, 2011 to May 31, 2012 in 2011/2012 season, so we calculated the number of newborn babies calculated in this season as the half of them

As shown in Figure 1, the RV vaccine coverage rates (RV1 and/or RV5) in Shibata City from epidemic seasons 2011/12 to 2014/2015 significantly increased every season, in contrast to all the seasons after the 2014/2015 season. Therefore, we refer to the former period as the transitional era and the latter as the plateau era. The average of vaccine coverage rates (73.0%) in the plateau era was significantly higher than that in the transitional era (44.9%) (*p* < .05).

*p < .05 (χ^2^ test).

**Figure 2.**
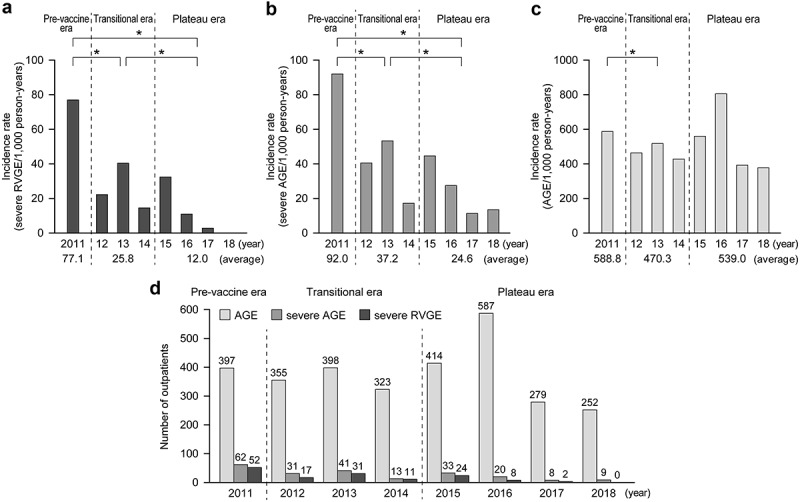
Incidence rates of severe RVGE (A), severe AGE (B), and AGE (C) among children younger than 3 years of age. The incidence rates were calculated based on the numbers of patients who visited the four clinics during each observation period (by May 31) and the number of children younger than 3 years of age who lived in Shibata City

The incidence rates, that is, per 1000 person-years, of severe RVGE, severe AGE, and AGE among children younger than 3 years of age during the three eras were calculated. The incidence rates of severe RVGE, among children younger than 3 years of age in the pre-vaccine introduction, transitional, and plateau eras were 77.1, 25.8, and 12.0, respectively (Figure 2A). The incidence rates of severe AGE in the pre-vaccine introduction, transitional, and plateau eras were 92.0, 37.2, and 24.6, respectively (Figure 2B). Lastly, the incidence rates of AGE in the pre-vaccine introduction, transitional, and plateau eras were 588.8, 470.3, and 539.0, respectively (Figure 2C). The prevalence rates of severe RVGE and severe AGE among patients in the plateau era were significantly lower than that in the pre-vaccine introduction and transitional eras (*p* < .05).

* *p* < .05 (χ^2^ test).

**Figure 3.**
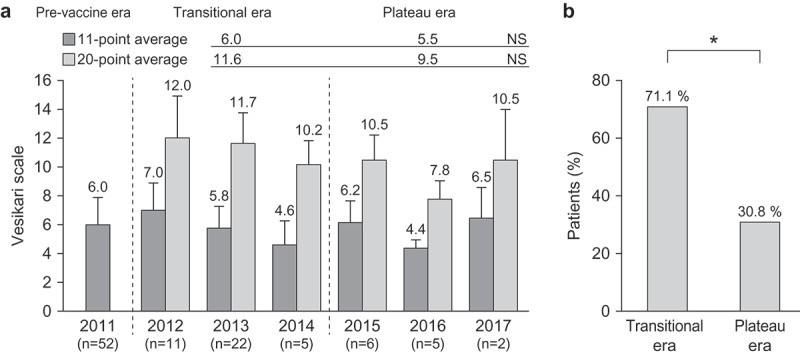
The 11-point and 20-point Vesikari scales in children with severe RVGE. (A) Averages of 11-point and 20-point Vesikari scales by year. (B) The proportion of the children who had more than 11 scores in two eras. They were calculated using the 20-point Vesikari scale

The Vesikari scale in children with severe RVGE is shown in Figure 3. There was no difference in 11-point and 20-point Vesikari scales by year (Figure 3A). Furthermore, the proportion of children who had more than 11 out of 20 points in the Vesikari scale was significantly lower in the plateau era than in the transitional era (*p* < .05) (Figure 3B).

*p < .05 (χ^2^ test).

Demographics and vaccination profiles of severe RVGE patients with previous RV vaccination history are provided in Table 1. There were 11 severe RVGE episodes observed in children who received the RV vaccine before the onset: 3 in the transitional era and 8 in the plateau era. All children, except one, were already vaccinated more than about 12 months before the onset of RVGE.

**Table 1.** Profiles of severe RVGE patients with vaccination history

		Transitional era			Plateau era							
		No. 1	No. 2	No. 3	No. 4	No. 5	No. 6	No. 7	No. 8	No. 9	No. 10	No. 11
Date of diagnosis		2013/2	2014/4	2014/5	2015/4	2015/5	2016/2	2016/2	2016/3	2016/5	2017/3	2017/5
Age in months		17	25	15	23	34	35	16	34	3	16	19
Sex		Male	Female	Male	Male	Male	Male	Female	Female	Male	Male	Male
Vaccine		Rotarix	Rotarix	RotaTeq	RotaTeq	Rotarix	Rotarix	Rotarix	RotaTeq	RotaTeq	Rotarix	Rotarix
Completed*		Yes	Yes	Yes	Yes	Yes	Yes	Yes	Yes	No	Yes	Yes
The length from last vaccination (months)		12	20	11	18	29	33	13	28	2	13	16
Severity score	20-point Vesikari Scale	11	-	11	8	10	9	6	8	7	13	8
	11-point Vesikari Scale	6	-	9	4	6	5	4	4	4	8	5

*“Completed” means Rotarix and Rotateq had been already administered the fixed frequency.

Genotypes of rotaviruses in feces collected from the study site and Shibata Hospital are summarized in Table 2. The majority of the genotypes in each epidemic year were G3P[8] in 2011, G1P[8] and G9P[8] in 2012, G1P[8] in 2013, G9P[8] in 2014, and G2P[4] in 2017.

**Table 2.** Genotypes of RV detected during the study period

	2011		2012		2013		2014		2015		2016		2017		2018	
	*n*	*(%)*	*n*	*(%)*	*n*	*(%)*	*n*	*(%)*	*n*	*(%)*	*n*	*(%)*	*n*	*(%)*	*n*	*(%)*
G1P[8]	1	5.9%	18	45.0%	39	88.6%	0	0%	0	0%	0	0%	0	0%	0	0%
G2P[4]	1	5.9%	1	2.5%	5	11.4%	1	16.7%	1	6.7%	2	66.7%	12	80.0%	0	0%
G3P[8]	14	82.4%	1	2.5%	0	0%	0	0%	0	0%	0	0%	2	13.3%	0	0%
G3P[9]	0	0%	0	0%	0	0%	0	0%	0	0%	0	0%	0	0%	1	33.3%
G9P[8]	1	5.9%	20	50.0%	0	0%	5	83.3%	14	93.3%	1	33.3%	1	6.7%	2	66.7%
Total	17		40		44		6		15		3		15		3	

**Figure 4.**
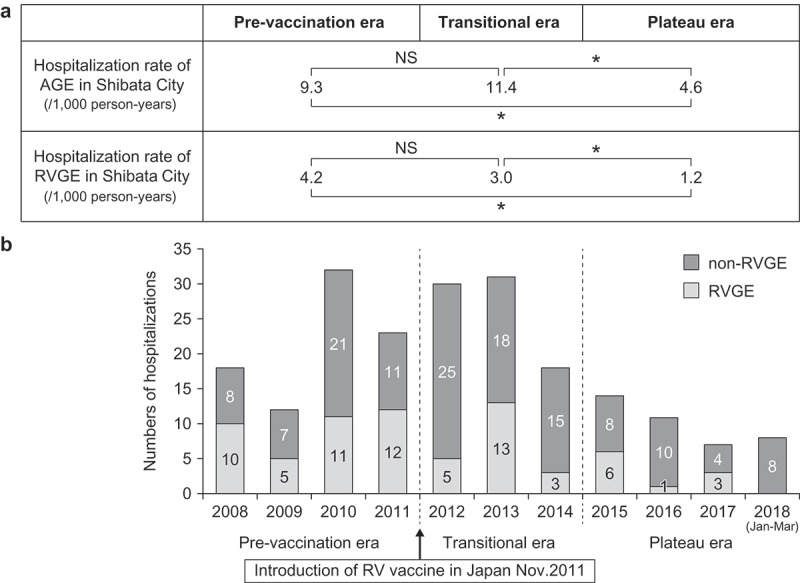
(A) Hospitalization rate of AGE in Shibata City (/1,000 person-years) and RVGE in Shibata City (/1,000 person-years). (B) Numbers of RVGE and non-RVGE hospitalizations (children younger than 3 years of age) who lived in Shibata City

The annual number of hospitalizations at Shibata Hospital due to RVGE and AGE that were collected retrospectively, the hospitalization rate of RVGE in Shibata City, and the proportion of RVGE among AGE hospitalization were compared among three periods: the pre-vaccine introduction (2008 to 2011), transitional (2012 to 2014), and plateau (2015 to 2018) eras. Figure 4A indicates the number of hospitalizations due to RVGE and non-RVGE by year. The hospitalization rate due to RVGE in Shibata City in the plateau era was significantly lower than that in the pre-vaccination and transitional eras, but the hospitalization rate due to AGE in the transitional era was significantly lower than that in the pre-vaccination era (*p* < .05 for all these comparisons). The hospitalization rate due to AGE in Shibata City was also significantly lower in the plateau era than in the pre-vaccination introductory and transitional eras (*p* < .05). (Figure 4B)

*p < .05 (χ^2^ test).

## Discussion

In this ecological study, we prospectively assessed severe RVGE occurrence and incidence among children of less than three years of age during hospitalization and in outpatient settings since the pre-vaccine introduction era. After our second report,^8^ vaccine coverage rates of RV1 and RV5 increased even further, and the incidence rates of severe RVGE in both hospitalization and outpatient settings were significantly lower than those we previously reported.

Many studies have reported RV vaccine effectiveness after the introduction of RV vaccination in national immunization programs.^12-17^ In almost all of them, the impact of RV vaccination was indicated in terms of RVGE hospitalizations or emergency department (ED) visits used in some hospital-based and primary practice-based studies conducted in other countries. For this reason, it is very important to estimate the number of outpatients with severe AGE accurately in Japan. Another important point is that the vaccine coverage rate was accurately calculated because the observation area was limited. In this study, significant reductions were observed in severe RVGE events at the clinics and the number of the patients with RVGE requiring hospitalization between the transitional and plateau eras. Hemming M et al. reported that hospitalizations or outpatient clinic visits due to RVGE were reduced by 76% and 81%, respectively, before and after the introduction of the National Immunization Programme (NIP) in Finland, considering a time frame of two years prior to and after introducing the RV vaccination in NIP.^17^ As a reference, the vaccine coverage rate in the period before and after the period of NIP was approximately 30% and more than 90%, respectively. In our study, there was no difference in hospitalization rate of patients with RVGE between the pre-vaccine and traditional eras, but there was a difference between the traditional and plateau eras. This difference could be due to the fact that, as already mentioned in the introduction, many children with severe AGE can go to a hospital and get admitted easily because of better access to a medical setting. That is, the inpatients selected were regarded as those with milder AGE than those in any other countries. Therefore, the efficacy of RV vaccine for inpatients was not clear when the coverage rate of RV vaccine was about 50%. Although a small increase in the vaccine coverage rates between the transitional and plateau eras was noted in our study, we considered that the increase corresponded to the significant reduction in hospitalizations as well as outpatient clinic visits due to RVGE. Typically, it is expected that further reduction of severe RVGE would occur after introduction of RV vaccine into the NIP in Japan.

We also only investigated the G and P genotypes of the Rotavirus samples collected in our research, as epidemic viruses may change by year and region in Japan.^18^ In our study, we could not observe a trend in the distribution of G and P genotypes in our regions.

Roczo Farkas et al. reported that dominant genotypes were influenced by the type of RV vaccine used in each state in Australia.^19^ Because both vaccines were introduced in Japan, there was no trend in dominant genotypes; however, new genotypes such as G12P[8] have become dominant in some areas recently.^20-23^ Therefore, future studies should investigate new genotypes. Furthermore, it was suggested that at least within 12 months from being vaccinated almost all children were protected from severe RV infection. Because it had been more than 12 months after receiving RV vaccine for almost of all the patients with RVGE, even those who needed intravenous injection received RV vaccine. However, there are some reports on unexpected genotypes such as G12P[8] RV genotype worldwide recently.^20^ Therefore, we should continue further research in the genotypes of RV.

There are some limitations of this surveillance study. First, severe RVGE was defined by the need of intravenous rehydration, according to all four pediatricians, which is subjective. Therefore, this definition should be evaluated objectively. However, we used the Vesikari scale to evaluate the severity of children’s conditions who suffered from severe RVGE, and no difference was observed between the seasons. We have also added the Vesikari scores for objectivity. Therefore, we considered that there were no significant differences between the decisions made by the four pediatricians based on their experience. Second, we only had data from one season of observation at four clinics for the pre-vaccination introductory era. Therefore, we cannot clarify any difference between pre-vaccination era and transitional or plateau era. However, we also investigated the number of RVGE hospitalizations between 2007 and 2010, as data in the pre-vaccination introductory era. As indicated in Figure 4, we can describe that there was a difference in the hospitalization rate of RVGE only between the pre-vaccination introductory and plateau eras. Therefore, if we have patient information for several years in the pre-vaccination era, the results might be different. Third, we did not estimate the safety strictly. In fact, there was no severe adverse event such as intussusception observed in our study. The incident rate of intussusception among children with RV vaccination is reported to be 1–6 excess cases with intussusception per 100,000 vaccinated infants,^24,25^ but we enrolled less than 1000 persons per year. Therefore, it was difficult to evaluate the rate of adverse even correctly in our research. Fourth, we concomitantly used two RV vaccines, Rotarix and RotaTeq, because both were licensed. However, both show similar efficacy for prevention from severe RVGE. Therefore, the results in our research might not have changed by the ratio of Rotarix and RotaTeq.^3^ The last limitation is insufficient economical effectiveness. However, the cost-effectiveness is influenced by the cost of vaccine,^26^ and there is no definitive opinion about the economic impact of RV vaccine in Japan, and thus, we should evaluate cost-effectiveness in the future.

In summary, significant reductions were observed in the incidence rates of severe RVGE cases, which required hospitalization and intravenous rehydration at the pediatric clinics in Shibata City, in correlation with the increase in vaccine coverage rates by more than 70% after the 2014/2015 season.

Our results suggest further reduction in severe RVGE rates after the introduction of a universal RV vaccination in Japan. RV vaccine will be adapted for universal use in Japan by next autumn. Our findings provide data for efficient vaccine use in the future.
